# Fear of childbirth and its associated factors among pregnant women in Dejen Woreda, East Gojjam Zone, Northwest Ethiopia: a community-based cross-sectional study

**DOI:** 10.1038/s41598-024-58855-5

**Published:** 2024-04-23

**Authors:** Chekol Alemu, Habitamu Wudu, Samuel Lakew

**Affiliations:** 1 Department of Statistics, College of Natural and Computational Sciences, Gambella University, Gambella, Ethiopia; 2 Departments of Midwifery, School of Public Health, College of Medicine and Health Sciences, Kurar Health Center, East Gojjam Zone, Dejen Woreda, Amhara Region Ethiopia

**Keywords:** Pregnant women, Wijma delivery expected questionnaire, Fear of childbirth, Health care, Health occupations, Medical research

## Abstract

Fears of delivery are the uncertainty and worry experienced before, during, and following labor. It hurts women's health and affects 5–40% of all moms globally. If not recognized, it could cause expectant mothers to feel alone and unsupported. Studies on this subject, however, are scarce at the woreda level. Therefore, this study amis to assess the prevalence and associated factors of fear of childbirth among pregnant women in Dejen Woreda, East Gojjam Zone, Northwest Ethiopia. A community-based cross-sectional study was conducted among 575 pregnant women selected by Cluster Sapling from December 15 to December 25, 2022. Data were gathered using a structured questionnaire that was presented by an interviewer. Data were entered using Epi-data version 3.1 and analyzed using SPSS Version 23 statistical software. Descriptive statistics and inferential statistics were done, and ordinary logistic regression was used to examine the associated factor for fear of childbirth. Finally, a P-value < 0.05 was used to determine statistical significance. Among the 575 pregnant women supposed to have participated, 560 agreed and participated in the survey, with a response rate of 97.4%. This study showed that 133(23.8%; CI 20.4–26.8) of the study participants had low fear of childbirth, 67(12%; CI (9.3–14.8 moderate, 217 (38.8%; CI 34.6–42.7) high, 143 (25.5%; CI 21.8–29.1) severe fear of childbirth. Having maternal age 18–24 (adjusted odds ratio/AOR = 1.6; 95% CI (1.1–2.3), p-value = -0.08), occupation daily laborer and other (AOR = 0.3,95%; CI 0.3, 0–74; p-value = 0.004),gestational age in third trimester (AOR = 1.9,95%; CI 1.1–3.4), p-value = 0.022) showed significant factor for a fear of childbirth. Maternal age, occupation, and third-trimester pregnancy were found to be significantly associated with fear of childbirth. Women should engage in special attention to keep them healthy by consistent monitoring during pregnancy. Healthcare providers should identify pregnant women with high fear of childbirth early, offer cognitive behavioral therapy, support psychological and physical well-being, provide early age and preventive measures, and use uniform instruments for assessing women's anxiety, promoting systematic reviews and longitudinal studies.

## Introduction

Fear of childbirth (FOC) is defined as feelings of uncertainty and anxiety before, during, or after childbirth, thinking about future labor and giving birth. Both empirical research and clinical practice have been more interested in tocophobia (extreme dread of delivery) during the past 30 years. Pregnant women's anticipatory delivery expectations are used to gauge their level of childbirth dread, whereas postpartum women's actual birthing experiences are used to gauge their level of fear^1^.

Experiences are used to gauge their level of fear^2–4^. Fear of childbirth is a specific fear in anticipation of future birth, ranging from inconsequential to very intense, and as feelings of unease and anxiety before, during, or following childbirth, as well as thinking about upcoming labor and delivery^5,6^. It may fall under the primary or secondary categories. Primary tocophobia is a woman's obsessive fear of delivery when she has never been pregnant before. In youth or the early stages of adulthood, fear of childbearing may begin. When a traumatic obstetric incident occurs during a prior pregnancy, secondary tocophobia, also known as a morbid fear of childbirth, develop^6^. Even though the Ethiopian government places a strong emphasis on maternal and child health, the country nevertheless has high rates of maternal and newborn mortality. Less attention has been paid to the psychological component because the health care system for women traditionally places more emphasis on physical health^7^.

Some women experience fear of childbirth, which can range from mild to severe. A serious fear of childbirth is a desire to avoid being pregnant, giving birth, or the worry of having it interfere with daily activities^8^. Tocophobia is a condition that affects 6–10% of expectant mothers and can result in several complications for both the mother and the baby, including prolonged labor, dystocia, fetal distress, and hypoxia, as well as postnatal depression and posttraumatic stress disorder^9^. In advance of a future birth, there is a distinct fear known as the fear of childbirth, which can be mild to quite strong. Fear of childbirth (FOC) in pregnant women can have a detrimental impact on their everyday lives, prolong the birthing process, delay the development of the mother-infant bond, and raise the risk of postpartum depression^10^. In prenatal psychology, delivery is an unknown, uncontrollable, and inevitable event connected to life and fear of death^2^.

Childbirth fear is common in both pregnant and postpartum women in developed countries^11^. Interventions for tocophobic women should focus on assisting them in managing their severe anxiety related to pregnancy and childbirth so that they may accept the uncertainties, and doubts associated with it. Treatments should gradually lessen stress-related factors to improve fetal adaptability and prevent the need for a cesarean section^12^. Malawi saw a reduction in FOC and an increase in childbirth self-efficacy because of the companion-integrated birthing preparation for fear, self-efficacy, and maternal support. Under World Health Organization (WHO) guidelines, every expectant woman should get psychological care to increase her capacity to give birth^8^.

Fears of Childbirth ranges from almost total absence of fear to extreme fear as the level of fear increases its consequences become sever^2,10,13^. Childbirth anxiety throughout pregnancy increases the likelihood of a difficult, protracted, and distressing labor. In addition, fear of Childbirth r has been associated with adverse postpartum mental health difficulties^14^. Many studies reported that FOC could be a reason for deciding to have a cesarean section for delivery when there is no medical cause for it. Higher childbirth fear during the third trimester of pregnancy increases the likelihood of choosing a cesarean section^15,16^. Over recent decades, the worldwide cesarean rate has continuously increased even though it is associated with higher complications^17^.

Even though maternity care is safe in high-income nations, fear of Childbirth r remains a common issue. 5–40% of mothers worldwide are impacted^18,19^. According to a study, the prevalence of fear of Childbirth ranges across six European nations, from 4.5 to 15.6%. In southern Ethiopia's Arba Minch town, a survey found that 25.3 and 24.5% of women, respectively, suffer from high degrees and severe degrees of dread^20^. Another study in Jinka Town, Southern Ethiopia, and West Wollega Zone shows childbirth fear affects 24.2% and 28.9% of pregnant mothers respectively^21^. Several studies have found that past pregnancy complications, experience of delivery complications, and the presence of strong social support increase the likelihood of experiencing childbirth fear^18,21,22^. Therefore, prompt and timely management of these complications and the promotion of maternal health are^11,23^. Therefore, one approach to dealing with this is to provide expectant moms with a safe environment in which to get both emotional and physical care from their families as well as from health institutions. The information needed to do so in Ethiopia is extremely scarce, and this problem has not yet been evaluated in the region where the current study was conducted. It is crucial to understand the scope and contributing elements of birthing anxiety to improve women's health. The findings from the study may have implications for midwives/health care providers and policymakers in terms of improving women's awareness and providing evidence-based practice. Therefore, this study aimed to assess the Prevalence and determinant factors of childbirth fear among pregnant women in Dejen Woreda, East Gojjam zone, northwest Ethiopia.

## Materials and methods

### Study design, setting, and period

A community-based cross-sectional study design was conducted at Dejen Woreda, East Gojjam Zone, Northwest Ethiopia from 15 to 25 December 2022.Dejen Woreda is one of the Woreda in the east Gojjam zone, Amhara region of Ethiopia. It is located 70 km from Debre Markos, 345 km from Bahir Dare, and 229 km from Addis Ababa. 24 kebeles total, 22 rural and 2 urban make up the woreda. According to the population projection, the total population in the woreda is expected to be around 128,784 (Female = 64,521), (Male = 64,263). A total of 1264 pregnant women were found in Dejen Woreda during the study period.

### Study population and recruitment criteria

The study population for this study was all pregnant women who lived at least six months in the picked from with selected kebeles of Dejen Woreda and available at the time of data collection in Dejen Woreda, East Gojjam Zone were included in the study. Pregnant mothers who were unable to respond to the questionnaire due to serious illness and health problems were excluded.

### Sample size determination

The sample size was calculated using a formula for a single population proportion considering a confidence interval of 95% (Z = 1.96), a margin of error of 5% (d = 0.05), and a prevalence of (p = 28.9%) of fear of childbirth from a previous study^23^.

$$n=\frac{{\left({{\text{Z}}}_{\frac{\mathrm{\alpha }}{2}}\right)}^{2}\times {\text{p}}(1-{\text{p}})}{({{\text{d}})}^{2}}={(1.96)}^{2}\frac{0.289(1-0.289)}{{(0.05)}^{2}}= 316.$$

After considering a 10% non-response rate and adding a design effect of 1.5 the final sample size was 575.

### Sampling procedure

A cluster sampling technique was used in selecting the study subjects. Accordingly, Dejen Woreda has 24 kebeles (The smallest administrative unit of Ethiopia, contained within a woreda.), of the total 24 Kebles, 10 Kebles were selected using a Simple random sampling technique. Secondly, a census was conducted to identify pregnant mothers in every ten Kebeles with the help of community health extension workers, Then Proportional allocation was applied to select the study population; finally, simple random sampling techniques were used to select the final 575 study participants.

### Variables and measurement

#### Sociodemographic variables

Maternal age, marital status, educational status, and occupation.

#### Obstetric related variables

gravidity/Parity, previous mode of delivery, previous birth outcome and obstetric complication, history of abortion, gestational age, ANC follow-up, pregnancy status, and current pregnancy complication (obstetric/medical).

### Social factors

Husband support and social support:—investigators measure the Husband support by asking them, had got support from your husband? YES or NO Response/Husband support present or Absent^24^ and, the level of social support also was assessed using the Oslo Social Support Scale (OSSS-3)^25^. This tool had three items, and the sum score ranged from three to 14. A total score of 3 to 8 is considered poor social support. A total score of 9 to 11 is considered moderate social support. Finally, a total score of 12 to 14 is considered strong social support.

### Fear of childbirth

33 item question that was used to assess the degree of fear of childbirth (FOC) according to the W-DEQ sum score is classified as low (≤ 37), moderate (38–65), high (66–84), and severe (≥ 85).

### Data collection

Six BSC Midwives and five health extension workers were recruited as data collectors and a health officer did the supervision. Training about the study's objectives and data collection procedures was given to the data collectors and the supervisor for one day. While conducting data collection, a health officer supervises the midwifery and the extension workers for the accurate use of data collector materials.

### Study instruments

Data were collected through an interviewer-administered structured questionnaire developed after reviewing previous studies. The questionnaire was first prepared in English then translated to Amharic and back-translated to English by language experts to maintain its consistency. The questionnaire had four components. These were Sociodemographic factors, obstetric-related factors, social support, and fear of childbirth (FOC). To assess the level of FOC, the Wijma Delivery Expectation and Experience Questionnaire (W-DEQ) version A was adopted. The W-DEQ is a six-point Likert-scale questionnaire with 33 items ranging from zero (not at all) to five (extremely), giving a minimum score of zero and a maximum score of 165. Furthermore, the level of social support was assessed using the Oslo Social Support Scale (OSSS-3)^25^. This tool had three items, and the sum score ranged from three to 14.

### Data quality management

A pretest was conducted on 5% of the sample size at Hagereselam Keble. Data collected for the pretest was analyzed and used for amending the data collection tool. Then reliability of the data collection tool was measured, data collection time was estimated and modifications such as logical order and rewriting items difficult to understand were made as well. The five-day training was given to data collectors and supervisors regarding the objective of the study, data collection tool, procedures, and how to approach respondents. Data quality, consistency, and completeness were maintained through daily collection and inspection.

### Data analysis

The Statistical Package for Social Science (SPSS) Version 23 was used to analyze the data after they had been entered using Epi-Data version 3.1. Descriptive analysis (frequencies, percentages,) was computed to explore socio-demographic, obstetric and other health-related characteristics and social support characteristics of study participants. Ordinary logistic regression was used to examine the association between fear of childbirth and explanatory variables. Variables with p-values of 0.25 in the bi-variable analysis were included in the multi-variable analysis. The outcome variable was on four scales based on fear of childbirth (low, modern, high, severe). Each independent variable was fitted in the model separately and checked for the assumption of ordinary logistic regression. Then those that passed the assumption of the model were fitted to predict the outcome variable. The odd proportionality assumptions of ordinal logistic regression were assessed using the parallel lines test. Values for the final model assumptions were as follows, model fitting information, goodness of fit (Pearson chi square), Deviance chi square, and Test parallel lines chi square. Finally, Variables with p-values less than 0.05 were considered statistically significant. Results from the final model were reported as an adjusted odds ratio (AORs) with a 95% confidence interval (CIs).

### Ethics approval and consent to participate

The Institutional Review Board (IRB) of Debre Makos University granted ethical approval, with reference number GURPGC/201/2015. The study participants were informed of the study's importance and goal. They were asked for their consent to participate before beginning the questionnaire, and they were told they could stop at any point without providing a reason if they changed their minds. They were also made aware that their information would be kept private. Participants' privacy was safeguarded. Finally, written informed consent was obtained from each participant before they began the study. All procedures were carried out in conformity with the necessary standards and laws (Declaration of Helsinki).

## Results

From 575 pregnant women supposed to be included, 560 agreed and participated in the survey, with a response rate of 97.4%.

### Socio-demographic characteristics

The mean age of the respondents was 25 (SD ± 3.2.) years, the majority of them 334 (59.6%) fell into the age group of 25–34 years. The vast majority of the respondents 493 (88%) were married, and about 127 (22.7%) of respondents attended primary education (Table 1).

**Table 1:** Socio-demographic characteristics of pregnant women in Dejen woreda, East Gojjam zone, Northeast Ethiopia, 2023(n = 560).

Variable	Category	Frequency	Percentage
Age	18–24	167	29.8
	25–34	334	59.6
	> = 35	59	10.5
Marital status	Single	45	8.1
	Married	493	88.0
	Divorced and widowed	22	3.9
Education	Non formal education	321	57.3
	Primary education	127	22.7
	Secondary education	40	7.1
	College and above	72	12.9
Occupation	Housewife	390	69.6
	Self employed	43	7.7
	Government employed	78	13.9
	Other laborer^@^	49	8.8

^@^Wood collecting, farmer, jobless, daily laborer, prolonged labor.

### Obstetric details of participants

Concerning respondent's obstetric characteristics, 65.9% were multigravida. Of these, the majority (80.5%) of them delivered by SVD. Additionally about 7.6% of multigravida and stillbirth during their previous childbirth, Moreover one-fifth (21.4%) of multigravida had obstetric complications in their previous pregnancy. Furthermore, the majority (89.9%) of the current pregnancies are planned pregnancy. Most (87.9%) of the pregnant women had ANC visits in their current pregnancy, and most of the participants (96.3%) preferred vaginal delivery. The majority (82.5%) of participants had support from their husbands during the current pregnancy (Table 2).

**Table 2:** Obstetric characteristics of pregnant women in Dejen woreda in, East Gojjam zone, Northeast Ethiopia, 2023 (n = 560).

Variables	Category	Frequency(N)	Percentage (%)
Gravid	Primigravid	191	34.1
	Multigravida	369	65.9
Parity (n = 369)	Nulliparous	17	4.6
	prim parous	163	44.2
	Multiparous	189	51.2
Previous mode of delivery (n = 369)	SVD	297	80.5
	Instrumental delivery	52	14.1
	C/S	20	5.4
Outcome of previous childbirth (n = 369)	Alive	341	92.4
	Still birth	28	7.6
History of abortion (n = 369)	Yes	62	16.8
	No	307	83.2
Complication in previous pregnancy/childbirth (n = 369)	Yes	79	21.4
	No	290	78.6
Complication in previous pregnancy/childbirth (n = 369), If yes	Vaginal bleeding	26	32.9
	Prolonged labor	44	55.7
	Pregnancy hypertension	7	8.9
	Other	2	2.5
Current pregnancy	Planned	464	82.9
	Unplanned	96	17.1
ANC follow up current pregnancy	Yes	492	87.9
	No	68	12.1
Preferred mode of delivery the current pregnancy	Vaginal delivery	539	96.3
	C/S	21	3.8
Pregnancy related complication the current pregnancy	Yes	55	9.8
	No	505	90.2
Pregnancy related complication the current pregnancy, If yes	Vaginal bleeding	8	14.5
	Hyper emesis gravidarium	28	50.5
	Hypertension	12	21.8
	Other	7	12.7
Medical illness/chronic disease the current pregnancy	Yes	22	3.9
	No	538	96.1
Medical illness/chronic disease the current pregnancy, If yes	Heart disease	5	22.7
	HIV	5	22.7
	Gestational diabetes mellitus	2	9.1
	HTN	10	45.5
Gestational age	First trimester pregnancy	56	10
	Second trimester pregnancy	301	53.8
	Third trimester pregnancy	203	36.3
Husband support	Yes	462	82.5
	No	98	17.5

### Social support of the respondents

**Figure 1:**
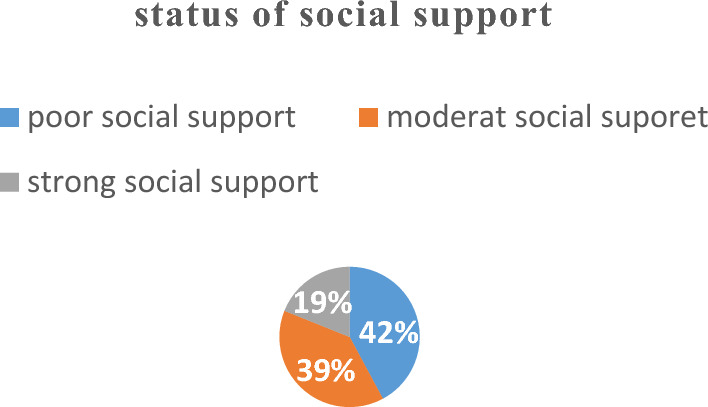
Social support among pregnant women pregnant women in Dejen Woreda, East Gojam Zone Northwest Ethiopia, 2023 (n = 560).

Regarding the respondents’ social support based on the Oslo social support scale, 236 (42.1) respondents, had poor social support scoring (Fig. 1).

### Prevalence of fear of childbirth

**Figure 2:**
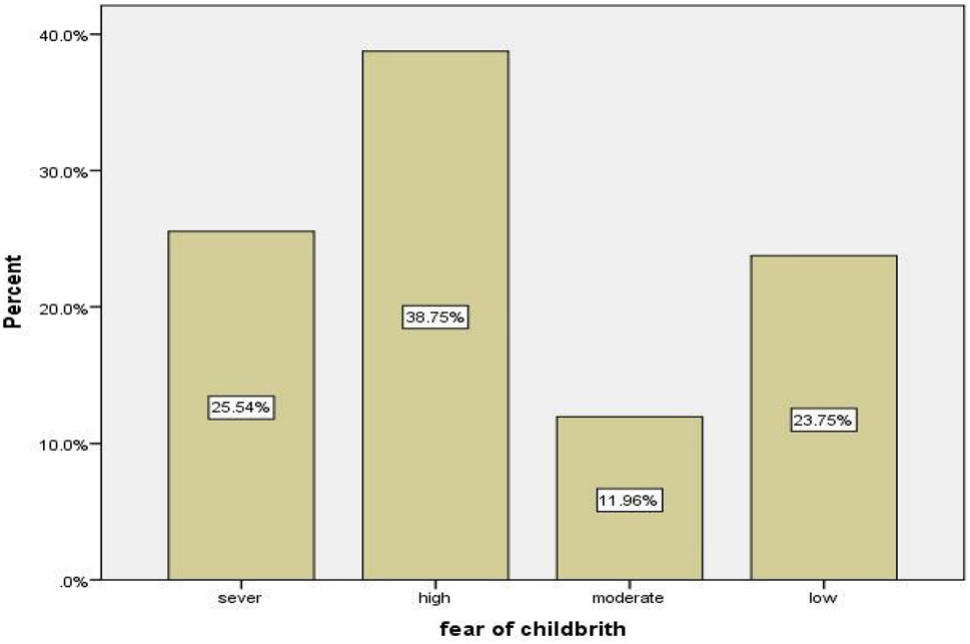
Prevalence of fear of childbirth among pregnant women in Dejen Woreda, East Gojam Zone Northwest Ethiopia, 2023 (n = 560).

Among all pregnant women, one-fourth (25.5%) had a severe level of childbirth fear. In addition, 23.8%, 12%, and 38.8% of pregnant women had low, moderate and high levels of childbirth fear respectively (Fig. 2).

### Associated factors for fear of childbirth

Univariable variable analysis showed Maternal age, occupation, Gestational age, ANC follow-up current pregnancy, any medical complication in the current pregnancy, and Husband support were factors associated with fear of childbirth (p-value 0.25) and added to multivariable ordinary logistic regression. In the multivariable ordinal logistic analysis maternal age, occupation and gestational age were significantly associated with FOC.

The odds of developing a severe level of fear of childbirth among pregnant women aged between 18 and 24 years was 1.6 times higher as compared to those women aged between 25 and 34 years (AOR: = 1.6, 95% CI 1.1, 2.3). The odds of developing a severe level of fear of childbirth among pregnant women in the other occupation categories were reduced by 70% as compared to Mother to Mother in the housewife categories(AOR = 3, 95% CI 3.31–5.74). Lastly, the odds of developing a severe level of fear of childbirth among pregnant women was 1.9 times higher than those in the first trimester (AOR = 1.9, 95%CI ( 1.1, 3.4) (Table 3).

**Table 3:** Ordinary logistic regression for factor associated with fear of childbirth (n = 560).

Variables	Sever	High	Modern	Low	COR (95% CI)	P- value	AOR (95% CI)
Age							
18–24 years	35	64	20	48	1.5 (1.1, 2.1)	0.008	1.6 (1.1,2.3) **
25–34 years^®^	94	131	42	67	1		1
≥ 35 years	14	22	5	18	1.4 (0.8, 2.3)	0.357	1.3 (0.8, 2.2)
Occupation							
Housewife^®^	92	143	50	105	1		1
Self-employed	13	16	9	5	0.6 (0.4, 1.1)	0.239	0.7 (0.4, 1.3)
Government employed	15	14	5	14	0.8 (0.5, 1.2)	0.479	0.9 (0.6, 1.3)
Others laborer	23	14	3	9	2.73(3.1,6.7)	0.004	3(3.31, 5.74)**
ANC							
Yes	119	195	57	121	1.5 (0.9, 2.4)	0.198	1.4 (0.8, 2.3)
No^®^	24	22	10	12	1		1
Current pregnancy complications							
Yes	21	18	6	10	1		1
No	122	199	61	123	1.7 (0.99, 2.8)	0.217	1.4 (0.8, 2.4)
Any current medical illness							
Yes^®^	11	26	2	3	1		1
No	132	211	65	130	2.7 (1.2, 6.1)	0.058	2.3 (0.97, 5.5)
Husband support							
Yes^®^	113	178	56	115	1		1
No	30	39	11	18	0.7 (0.5, 1.1)	0.323	0.8 (0.5, 1.2)
Social support							
Low^®^	25	34	21	26	1		1
Moderate	54	93	42	29	1.31 (1.1, 2.1)	0.08	0.6 (0.75,2.3)
Strong	64	90	4	78	0.92 (0.8, 2.3)	0.357	1.65 (0.8, 2.2)
Gestational age							
First trimester^®^	21	21	5	9	1		1
Second trimester	70	126	37	25	1.8 (1.03, 3.0)	0.059	1.7 (0.97, 2.9)
Third trimester	52	70	25	56	1.9 (1.1, 3.3)	0.022	1.9 (1.1, 3.4)**
Intercept1/low FOC							0.0680
Intercept2/moderate FOC							1.715
Intercept3/high level of FOC							2.345

Dependent variable: fear of childbirth.

**Significant at p < 0.05.

^®^Reference ANC Antenatal care, *GA* Gestational age.

## Discussion

This community-based cross-sectional study was conducted to assess the fear of childbirth and associated factors among pregnant women in Dejen Woreda, East Gojjam Zone, and Northwest Ethiopia. Since almost all pregnant women had some degree of fear of childbirth(low, moderate, high and server), we used As comparison a WDEQ sum score of 85 or more as women having a severe degree fear of childbirth from other degree of fear of child Birth^26^ and the discussion was based on this cut-off point. This study found that 23.8%, 12%, 38.8%, and 25.5% of pregnant women had low, moderate, high, and severe levels of fear of childbirth, respectively. In contrast to a study conducted in Thailand, where the prevalence was low (18.5%), moderate (64.9%), high (16.1%), and severe (0.7%)^20^. This discrepancy could result from variations in sample sizes and participant selection practices. Other studies using W-DEQ ≥ 85 reported a prevalence of severe childbirth fear was 5.3 percent and high FOC was 36.7 percent in Ireland, and Norway^27,28^. In this study sever degree of fear of childbirth is Five times higher than the study conducted in Ireland (5.3% sever FOC) and Norway^27,28^ and On the other hand, in this finding the prevalence of sever FOC is 36 times higher than that of a study conducted in Thailand, in which the prevalence of sever FOC was found to be 0.7%^20^. The variation in the prevalence of FOC may be related to variations in the quality of prenatal and delivery care. Furthermore, sociocultural variations may account for variations in the prevalence of childbirth fear. Different cultures may influence people's opinions and attitudes regarding childbirth in different ways^29,30^. In this Study Saver degree of FOC is similar to study conducted in Arba Minch town, southern Ethiopia (24.5% sever FOC)^24^ and study conducted in which shows the prevalence of severe levels of fear of childbirth is 20%^31^. In addition, our finding is three times higher than the reported prevalence (8.0%) of severe fear of childbirth in Kenya^32^. This study also examined variables associated to fear of childbirth. Maternal age; occupation, and Gestational age were significantly associated with fear of Childbirth. Pregnant women who had previously other labor were three times more likely to suffer fear during childbirth than those who had not. This might suggest that these mothers consider themselves economically poor and at the same time fail to care for their children, they are temporary workers and family influence and This result is in line with research from six European nations—Belgium, Iceland, Denmark, Estonia, Norway, and Sweden—that discovered a strong correlation between fear of delivery and other occupations^19^ and it is also consistent with the Study conducted in West Wollega Zone^23^. Age from 18 to 24 years (AOR = 1.6; CI 1.1–2.3; P = 0.008) this might be due to mothers' intention to keep their posture safe, psychological immaturity about childbirth, freely use of their age, economical factor and being prime gravidae Numerous earlier research have identified maternal age and gestational age as risk factors for FOC which is similar with our finding^19,33^. Third trimester, also had a higher significance association (AOR = 1.9; 95% CI 1.1–3.4); p = 0.022) with the FOC. This might suggest that these mothers think as it takes prolonged time and painful episode during delivering, end up with death, the early stages of adulthood, fear of child bearing. But, the study conducted in Kenya indicates that no significant Association between FOC and socio-demographic variables including maternal age, gestation age, and occupation^32^.

Bringing fear of childbirth into the light as a problem among Ethiopian women may be taken as the strength of the study as most of the people including the women in the country themselves have not been considering the situation as a problem. But the limitation Cross-sectional study, design it does not show a true cause-and-effect relationship between the dependent and independent variables and the data collection process was primary data collection; it was tiresome to get those pregnant women in their kebele and the other limitation of this study was the inability to utilize the tool to evaluate certain characteristics, such as the history of abuse or violence during pregnancy, Residence, history of abortion which may have a significant association with the FOC.

## Conclusion

In the study setting, there is high level fear of childbirth. The variables that were shown to be substantially linked to fear of childbirth were maternal age, occupation, and Gestational age. Pregnant women between the ages of 18 and 24 years, who work as laborers, and who is third trimester of pregnancy should receive further attention and counseling. To reduce further FOC difficulties, it is critical that health care providers identify pregnant women who exhibit high level of fear of childbirth early in the pregnancy and offer cognitive behavioral therapy as well as support for their psychological and physical well-being. It is also recommended that family planning and ANC clinicians provide suitable information regarding early age and preventive measures. Given that Ethiopia is a multiethnic nation. In order to obtain comparable results, we also advise researchers everywhere to assess women's anxiety about giving birth using uniform instruments. Systematic reviews and/or longitudinal studies are further encouraged.

## Data Availability

The datasets used and/or analyzed during the current study are available from the corresponding author upon reasonable request.

## Abbreviations

ANC: Antenatal care
AOR: Adjusted odds ratio
CD: Caesarean delivery
CI: Confidence interval
COR: Crude odds ratio’s
EPDS: Edinburgh postnatal scale
FOC: Fear of Childbirth
IAVD: Instrumental assisted vaginal delivery
NGO: Nongovernmental organization
OSSS: Oslo social support scale
PP-PTSD: Postpartum posttraumatic disorder
SD: Standard deviation
SPSS: Statistical package for social sciences
SVD: Spontaneous vaginal delivery
WDEQ: Wijma delivery expectancy/Experience questionnaire
